# Taxonomic notes on *Lasioglossum (Lasioglossum) subopacum* (Smith) and *L. (L.) okinawa* Ebmer et Maeta (Hymenoptera, Halictidae) from Asia

**DOI:** 10.3897/zookeys.143.2077

**Published:** 2011-11-01

**Authors:** Ryuki Murao

**Affiliations:** 1 Entomological Laboratory, Faculty of Agriculture, Kyushu University, Fukuoka, 812–8581 Japan

**Keywords:** Hymenoptera, Halictidae, *Lasioglossum*, Asia, taxonomy

## Abstract

*Lasioglossum (Lasioglossum) subopacum* (Smith) is recorded from the Korean Peninsula for the first time. *Lasioglossum (Lasioglossum) okinawa* Ebmer et Maeta from Japan is ranked to a subspecies of *Lasioglossum (Lasioglossum) subopacum* judging from the characteristics of the male. The male of *Lasioglossum (Lasioglossum) subopacum* *okinawa* is described for the first time. Some bionomical notes of both subspecies are presented.

## Introduction

The halictine bee subgenus *Lasioglossum* s. str. Curtis, 1833 (Halictidae: Halictinae) is morphologically characterized by the second submarginal crossvein of female fore wing as strong as the first, and the female inner hind tibial spur serrate or pectinate with five or more teeth. This subgenus is mainly known from the Holarctic Region with 111 species recorded in the Palaearctic Region. Two of them, *Lasioglossum (Lasioglossum) subopacum* (Smith, 1853) and *Lasioglossum (Lasioglossum) okinawa* Ebmer et Maeta, 1999 are known to occur in Asia: the former from eastern to southeastern Asia (Pesenko 2006), and the latter only from the Ryukyu Islands, southwestern Japan (Ebmer and Maeta 1999). The latter taxon was originally described based on only female specimens. In the course of my study of Asian halictid bee fauna, I have been examined extensive series of specimens collected particularly from eastern Asia. Through careful examination, I have found *Lasioglossum subopacum* from the Korean Peninsula (South Korea) for the first time, as well as the previously undescribed males of *Lasioglossum okinawa*. In addition, I found that the male of *Lasioglossum okinawa* cannot be clearly separated from *Lasioglossum subopacum*, so I concluded that *Lasioglossum okinawa* should be properly treated a geographical race, a subspecies of *Lasioglossum subopacum*. In the present paper, I report the new taxonomic notes of *Lasioglossum subopacum* including the male description of *Lasioglossum subopacum okinawa* and some bionomical notes of both subspecies.

## Material and methods

This study is based on the specimens deposited in the following institutions, which are referred to in the text by the following abbreviations: ELKU, Entomological Laboratory, Faculty of Agriculture, Kyushu University, Fukuoka, Japan; EBSU, Prof. Emeritus Yasuo Maeta’s collection, deposited in the Division of Environmental Biology, Faculty of Life and Environmental Science, Shimane University, Matsue, Japan; BPBM, Maa’s collection borrowed from the Bernice P. Bishop Museum, Honolulu, Hawaii, USA; MNHAH, the late Dr. Shoichi F. Sakagami’s collection, deposited in the Museum of Nature and Human Activities, Hyogo, Sanda, Japan; and without acronym, my private collection, now deposited in the ELKU.

Terminology and style used in the description follow McGinley (1986), Murao and Tadauchi (2007). Abbreviations used in the text are as follows: Fn, nth antennal flagellomere; IS, interspaces between punctures; PP, punctures; Sn, nth metasomal sternum; Tn, nth metasomal tergum. The scientific names of flowering plants visited by bees are cited from Yonekura and Kajita (2003–).

**Figures 1–6.**
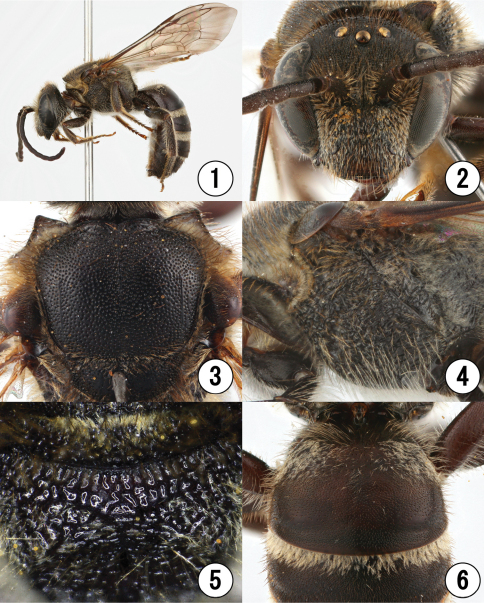
Male of *Lasioglossum (Lasioglossum) subopacum okinawa* Ebmer et Maeta **1** lateral habitus **2** head in frontal view **3** mesoscutum **4** mesosoma in lateral view **5** propodeal dorsum **6** first metasomal tergum.

Comparative material examined. *Lasioglossum (Lasioglossum) occidens* (Smith, 1873): 1♂, Rifu–cho, Miyagi Pref., Honshu, Japan, 14. VIII. 1979 (K. Goukon, MNHAH, illustrated in Fig. 12); 1♂, Kusasenri, Choyo–son, Aso–gun, Kumamoto Pref., Kyushu, Japan, 11. IX. 2004 (T. Sugimoto, illustrated in Fig. 16); 1♂, Mt. Ten–zan, Kyuragi–machi, Saga Pref., Kyushu, Japan, 13. VIII. 2004 (T. Sugimoto, illustrated in Fig. 17). *Lasioglossum (Lasioglossum) sakishima* Ebmer et Maeta, 1999: 1♂, Yonehara, Iriomote–jima, Okinawa Pref., Ryukyus, Japan, 18. VI. 1972 (O. Tadauchi, ELKU, illustrated in Figs 13, 18); 1♂, Ohtomi, Iriomote–jima, Okinawa Pref., Ryukyus, Japan, 23. V. 2003 (T. Mita, illustrated in Fig. 19).

## Taxonomy

### Lasioglossum (Lasioglossum) subopacum subopacum

(Smith, 1853)

http://species-id.net/wiki/Lasioglossum_subopacum_subopacum

Figs 11 20, 22

Halictus subopacus  Smith, 1853, Cat. Hym. Brit. Mus., 1: 63 [Syntype: Natural History Museum, London, United Kingdom; ♀, Foo–cho–foo (now Fuzhou, Fujian Prov.), north China]; Smith, 1873, Trans. ent. Soc. London, 1873: 200 [in list]; Dalla Torre, 1896, Cat. Hym., 10: 85; Cockerell, 1909, Ann. Mag. nat. Hist., (8)4: 316 [♀, in key]; Cockerell, 1919, Ann. Mag. nat. Hist., (9)3: 123; Wu, 1941, Cat. Ins. Sin., 6: 273; Hirashima, 1957, Sci. Bull. Fac. Agr. Kyushu Univ., 16(1): 20.
Halictus chinae  Strand, 1910, Berl. ent. Zeitschr., 54: 182 [Syntypes: Museum für Naturkunde an der Humboldt Universität zu Berlin, Germany; 2♀, Tsingtau (now Qingdao, Shandong Prov.), China]; Strand, 1915, Ent. Mitt., 4: 63 [in list]; Blüthgen, 1926, Deutsch. ent. Zeitschr., 1925: 396 [Synonymy]; Wu, 1941, Cat. Ins. Sin., 6: 272.
Halictus horishensis  Cockerell, 1911, Ann. Mag. nat. Hist., (8)8: 662 [Holotype: U. S. National Museum of Natural History, Smithsonian Institution, Washington, DC, USA; ♂, Horisha, Taiwan]; Ebmer, 1980, Linzer biol. Beitr., 11(1): 501 [Synonymy].
Halictus perangulatus  Cockerell, 1911, Ann. Mag. nat. Hist., (8)8: 663 [in key], 666 [Syntype: Museum für Naturkunde an der Humboldt Universität zu Berlin, Germany, 7♀, Taiwan]; Blüthgen, 1922, Deutsch. ent. Zeitschr., 1922: 63 [Synonymy].
Halictus baguionis  Crawford, 1918, Proc. ent. Soc. Washington, 19: 170 [Holotype: U. S. National Museum of Natural History, Smithsonian Institution, Washington, DC, USA; ♀, Luzon, Bagua, Philippines]; Blüthgen, 1926, Deutsch. ent. Zeitschr., 1925: 416 [Synonymy].
Lasioglossum (Sericohalictus) subopacum : Pesenko, 1986, Trudy Zool. Inst. Akad. Nauk SSSR, 159: 137 [in key].
Lasioglossum (Leuchalictus) subopacum : Pesenko, 2006, Zoosyst. Ross., 15(1): 159.
Lasioglossum (Lasioglossum) subopacum : Ebmer, 1980, Linzer biol. Beitr., 11(1): 500, 501; Sakagami & Tadauchi, 1995, Esakia, 35: 183, Fig. 16; Ebmer, 1998, Linzer biol. Beitr., 30(1): 410; Ebmer & Maeta, 1999, Linzer biol. Beitr., 31(1): 230, Figs 7–9.

#### Diagnosis.

This species is divided into two subspecies, one of which is newly relegated to a subspecies of *Lasioglossum subopacum* as treated below. The nominotypical subspecies is separated from the ssp. *okinawa* by only the female characteristic that propodeum, and T1 basally with dense and thick yellowish tomentose as in Figs 20, 22. In male, both subspecies cannot clearly separate. This subspecies is separated from the other Korean *Lasioglossum* s. str. species in having the combination of following characters: the mesoscutum reflexed upward in both sexes and densely transversely rows on medio-anterior margin in female; the female propodeum and T1 with dense yellowish tomentose; the shape of hair tufts on male S6 and the gonostylus as in Fig. 11; and the male genitalia without ventral retrorse.

#### Distribution.

China (north and southeastern areas), Taiwan, Vietnam, Philippines, and Korean Peninsula (south).

#### Flight record.

Female: March to December. Male: May to October.

#### Flower record.

In Korea, this species visited the following six species of flowering plants. Apiaceae: *Angelica miqueliana*. Asteraceae: *Aster yomena*; *Sonchus arvensis*. Brassicaceae: *Brassica* sp. Caprifoliaceae: *Lonicera* sp. Rosaceae: *Crataegus* sp.

#### Specimens examined.

[SOUTH KOREA] 2♀1♂, Cheju Is., 17. X. 2005 (O. Tadauchi and R. Murao), 22. X. 2005 (O. Tadauchi); 1♀, Myeong–do–am, 400–600m, Cheju city, Cheju Is., 15. IX. 1998 (O. Tadauchi, ELKU); 2♀, Pijarim Forests, Pukcheju–gun, Cheju Is., 24. IV. 1997 (O. Tadauchi and J. C. Paik, ELKU); 1♂, Pijarim, Pukcheju–gun, Cheju Is., 15. IX. 1998 (J. C. Paik, ELKU); 1♂, Kwanumsa, 500m, Cheju city, Cheju Is., 14. IX. 1998 (O. Tadauchi, ELKU); 8♀, KwangNung, Pocheon-shi, Gyeonggi–do, 19. V. 1992 (O. Tadauchi, ELKU).

[CHINA] Hunan Prov.: 1♀, Yuanling, 7. V. 1939 (T. C. Maa, BPBM); 1♀ Changteh, Yangshan, 11. X. 1938 (T. C. Maa, BPBM). Fujian Prov.: 1♀, Changting city, 3. VI. 1940 (T. C. Maa, BPBM); 12♀, Chungan, Bohea Hill, 11. VIII. 1939 (T. C. Maa, BPBM), 25. IX. 1939 (T. C. Maa, BPBM), 30. IX. 1939 (T. C. Maa, BPBM), 7. X. 1939 (T. C. Maa, BPBM), 24. IV. 1940 (T. C. Maa, BPBM), 6. V. 1940 (T. C. Maa, BPBM), 11. VIII. 1943 (T. C. Maa, BPBM); 1♀, Kienyang, Nwangkeng, 30. VII. 1943 (T. C. Maa, BPBM); 1♀, Kienyang, Liutun, 6. VII. 1942 (T. C. Maa, BPBM); 3♀ and 1♂, Shaowu city, 26. V. 1943 (T. C. Maa, BPBM), 11. IX. 1943 (T. C. Maa, BPBM), 8. XII. 1941 (T. C. Maa, BPBM), 23. XII. 1941 (T. C. Maa, BPBM, illustrated in Figs 20, 22); 1♀, Shaowu, 9. VII. 1942 (T. C. Maa, BPBM); 2♀, Shaowu, Shuipeichieh, 7. VII. 1941 (T. C. Maa, BPBM), 20. VII. 1941 (T. C. Maa, BPBM); 7♀, Shaowu, ShuiPeiKai, 16. III. 1942 (T. C. Maa, BPBM), 26. III. 1942 (T. C. Maa, BPBM), 13. V. 1943 (T. C. Maa, BPBM), 1. VI. 1943 (T. C. Maa, BPBM), V. 1945 (T. C. Maa, BPBM), 1♀, Shaowu, Tachuland, 25. IV. 1943 (T. C. Maa, BPBM); 2♀, Yungan, 19. X. 1940 (T. C. Maa, BPBM), 22. IV. 1941 (T. C. Maa, BPBM).

### Lasioglossum (Lasioglossum) subopacum okinawa

Ebmer & Maeta, 1999 stat. n.

http://species-id.net/wiki/Lasioglossum_subopacum_okinawa

Figs 1 10 14, 15 21, 23

Lasioglossum (Lasioglossum) okinawa  Ebmer et Maeta, 1999, Linzer biol. Beitr., 31: 230–233 [Holotype: EBSU; ♀, Okinawa–jima, Okinawa Pref., Japan)]; Ikudome, 1999, Ident. Guide Aculeata Nansei Is., Jap.: 746.

#### Diagnosis.

This subspecies is separated from the ssp. *subopacum* by the female propodeum and T1 basally with sparse and thin whitish tomentose as in Figs 21, 23. In Japan, it is closely similar to *Lasioglossum (Lasioglossum) occidens* (Smith, 1873) and *Lasioglossum (Lasioglossum) sakishima* Ebmer et Maeta, 1999. However, it is separated from the former by the mesoscutum reflexed upward in both sexes and densely transversely rows on medio-anterior margin in female, the T1 basally with whitish tomentose tufts in both sexes  (Figs 6, 23), the shape of hair tufts on male S6 (Fig. 15), and the gonostylus narrowly rounded apically as in Fig 10; from the latter by the basal elevation of male labrum broadly rounded (Fig. 14), the sculpture on female mesoscutum as stated above, the shape of both hair tufts on male S6 and gonostylus. In contrast, in *Lasioglossum occidens*, mesoscutum flat in both sexes and densely coarsely punctate on medio-anterior margin in female, T1 without tomentose hair tufts in both sexes as in Fig. 16, hair tufts on male S6 as in Fig. 17, and gonostylus broadly rounded as in Fig. 12; in *Lasioglossum sakishima*, basal elevation of male labrum small and rounded as in Fig. 18, female mesoscutum reticulate–punctate on anterior margin, shape of hair tufts on male S6 as in Fig. 19, and gonostylus truncate apically as in Fig. 13.

#### Description of male (new to science).

Body length 7.0–8.7mm, wing length 6.0–7.1mm (n=5).

Color. Body black except on the following parts: mandible apical half reddish brown; tegula blackish brown translucent; tibial spur yellow; posterior margin of metasomal terga narrowly brown translucent. Wings nearly transparent; veins and pterostigma blackish brown.

Pilosity. Mostly whitish; pale brown on mesosoma dorsally; mesoscutellum and metanotum mixed with blackish brown hairs. Head with sparse short fine branched hairs, and mixed with moderately dense tomentose on lower paraocular area. Hairs on mesosoma finely branched except on the following parts: dorsal, lateral surface, and around lateral lobe of pronotum with dense tomentose. T1 (Fig. 6) basally with a pair of tomentose tufts, however sometimes disappear. Disc on T2–5 with moderately dense short and simple hairs. Basal hair bands on metasomal terga present on T2–4 or T2–3. Apical fimbriae on metasomal terga absent. Acarinarium absent. S6 with well–formed, distinctive hair tufts as in Fig. 15.

Structure. Head nearly as long as wide; head length/width ratio 1.0–1.01 (n= 5). Vertex flat medially. Distance between lateral ocelli nearly as long as that between lateral ocellus and compound eye. Frons and paraocular area with reticulate–punctate, dimly shiny. Supraclypeal area slightly convex in lateral view, dimly shiny, with reticulate–punctate; IS with distinct tessellation. Clypeus 1.5× distance between lower rim of antennal socket and upper margin of clypeus; nearly flat, with moderately dense PP; IS nearly smooth. Basal area of labrum 2× as wide as long; basal elevation weakly developed, broadly rounded; distal process absent; labral fimbriae acutely pointed at apex. Mandible edentate. Hypostomal carina moderately developed; its anterior angle obtuse. Postgena slightly depressed, with distinct lineoration. Scape length 0.5–0.6mm (n= 5), F2 2.2× F1.

Pronotal dorsolateral angle acute, moderately projecting; pronotal lateral ridge incomplete, interrupted by oblique lateral sulcus; lower portion of lateral ridge inconspicuous, narrowly rounded. Mesoscutum (Fig. 3) with oily–dull luster, dense PP excluding anteriorly, IS smooth; its anterior margin weakly reflexed upwards, with reticulate–punctate. Mesoscutellum marginally and longitudinally with dense PP. Metanotum and mesepisternum coarsely rugulose. Propodeum coarsely rugulose; propodeal dorsum (Fig. 5) 0.7× mesoscutellum, and nearly as long as metanotum; shield marginally with lateral carina that not reaching to apical margin on dorsal surface. Basitibial plate of hind leg carinate marginally. Inner hind tibial spur without distinct teeth.

**Figures 7–13.**
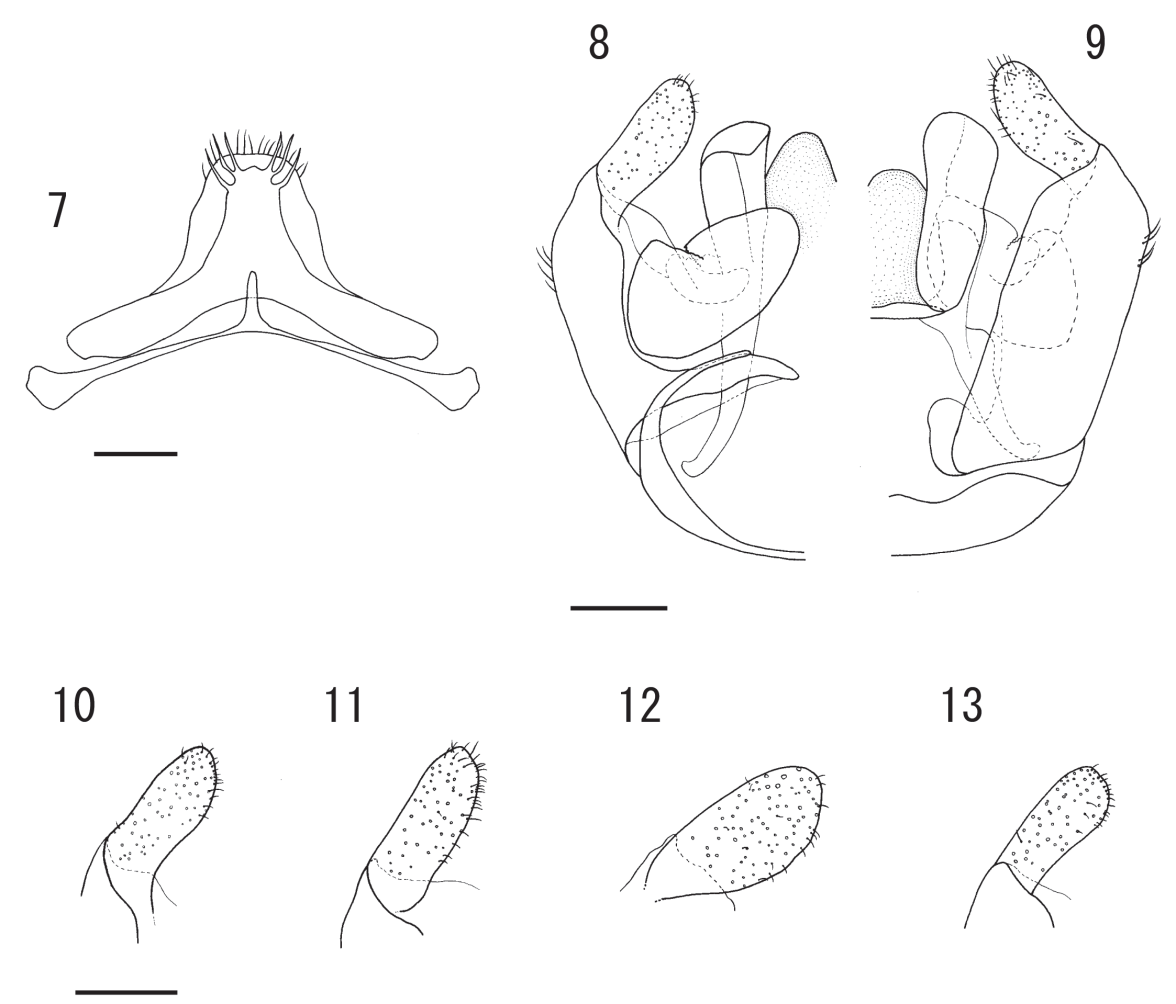
**7-10** Male of *Lasioglossum (Lasioglossum) subopacum okinawa* Emer et Maeta **7** seventh and eighth metasomal sterna **8** genitalia in ventral view **9** genitalia in dorsal view **10–13** gonostylus of genitalia in ventral view **11** Male of *Lasioglossum (Lasioglossum) subopacum subopacum* (Smith) **12** Male of *Lasioglossum (Lasioglossum) occidens* (Smith) **13** Male of *Lasioglossum (Lasioglossum) sakishima* Ebmer et Maeta Scale bars: 0.2mm.

Metasomal terga with oily–dull luster. T1 with weak lineolation over entire surface, medially and apically with dense PP. T2–3 with dense PP over entire surface; IS weakly lineolate over entire surface. T4–5 similar to IS of T2–3. S7–8 (Fig. 7): S7 with short and slender median process; S8 without median process. Male genitalia as in Figs 8–10. Gonobase ventral arm ring–shaped, and not connected to each other at apical ends: bottom nearly flat. Gonocoxite smooth. Gonostylus simple and flat, butter knife–like apically. Ventral retrorse lobe absent.

#### Variation.

Male clypeus black over the entire surface, or with a small yellow spot on lower margin.

#### Distribution.

Japan (central Ryukyus: Amami–ôshima, Okinawa–jima, Kume–jima).

#### Flight record.

Female: April to November. Male: May to October.

#### Flower record.

This species visited the following six species of flowering plants. Apiaceae: *Foeniculum vulgare*. Asteraceae: *Bidens pilosa* var. *radiata*; *Solidago altissima*. Euphorbiaceae: *Mallotus japonicus*. Polygonaceae: *Fallopia japonica* var. *japonica*; *Persicaria longiseta*.

#### Collecting site.

So far as the author’s survey in both Amami–ôshima and Okinawa–jima, this species was mainly collected from around subtropical forest at mountain areas, but in Kume–jima from open land such as cultivated area.

#### Specimens examined.

**Figures 14–19.**
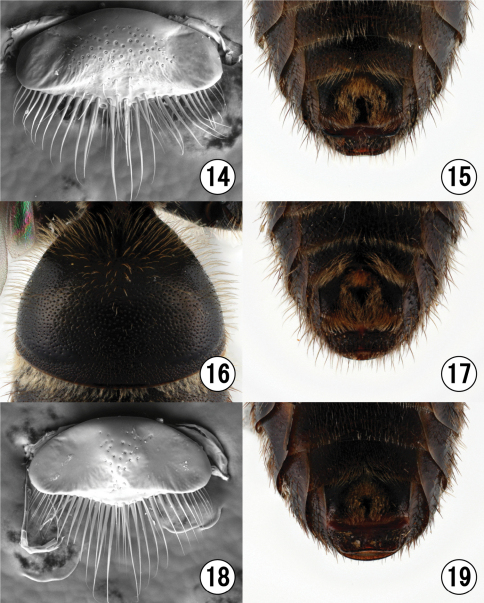
**14–15** Male of *Lasioglossum (Lasioglossum) subopacum okinawa* Ebmer et Maeta. **16–17** Male of *Lasioglossum (Lasioglossum) occidens* (Smith) **18–19** Male of *Lasioglossum (Lasioglossum) sakishima* Ebmer et Maeta **14, 18** labrum **16** first metasomal tergum **15, 17, 19** distal parts of metasomal sternum.

Holotype: ♀, Mt. Yonaha–dake, Okinawa–jima, Okinawa Pref., Japan, 7. VII. 1998 (Y. Maeta, EBSU). [JAPAN] [Ryukyus] Kagoshima Pref.: 1♀16♂, Mt. Yuwan–dake, Uken–son, Amami–ôshima, 14. X. 2004 (R. Murao, 1♂ illustrated in Fig. 1, 1♂ in Fig. 4, 1♂ in Figs 7–10, 1♂ in Fig. 15); 1♂, Mt. Yuwan–dake, alt.500m, Amami–ôshima, 17. VII. 1963 (C. M. Yoshimoto, ELKU); 2♂, Mt. Yuwan–dake, 24. VII. 1963 (Y. Hirashima, ELKU), 29. VII. 1963 (Y. Hirashima, ELKU); 8♀2♂, Shinmura, Amami–ôshima, 23. VII. 1954 (S. Ueda, ELKU; S. Miyamoto and Y. Hirashima, ELKU), 29. VII. 1954 (S. Ueda, ELKU); 1♀, Yuwan, Amami–ôshima, 31. VII. 1963 (Y. Hirashima, ELKU); 3♀, Santarou–toge, Sumiyou–son, Amami–ôshima, 15. X. 2004 (R. Murao, 1♀ illustrated in Figs 21, 23); 1♀, Yakkachi, Sumiyou–son, Amami–ôshima, 19. VII. 1933 (T. Esaki and K. Yasumatsu, ELKU). Okinawa Pref.: 3♀ (paratypes), same data as the holotype; 1♀1♂, 60–180m, Izumi, Motobu, Okinawa–jima, 22. V. 1982 (S. Ikudome, ELKU, 1♂ illustrated in Figs 2, 3, 5, 6); 1♂, Mt. Yonaha–dake, Kunigami–son, Okinawa–jima, 14. VI. 2002 (Y. Maeta, EBSU); 14♀, Hedo, Okinawa–jima, 5. IV. 1979 (K. Ohara, ELKU); 1♀, Nago, Okinawa–jima, 7. IV. 1979 (K. Ohara, ELKU); 1♀, Mt. Katsuu–dake, Nago–shi, Okinawa–jima, 3. XI. 2004 (R. Murao); 6♀3♂, Gima, Kume–jima, 27. V. 2003 (R. Murao); 3♀3♂, Zenda, Kume–jima, 27. V. 2003 (R. Murao, 1♂ illustrated in Figs 4, 14); 1♀, Nakadomari, Kume–jima, 26. V. 2003 (R. Murao).

**Figures 20–24.**
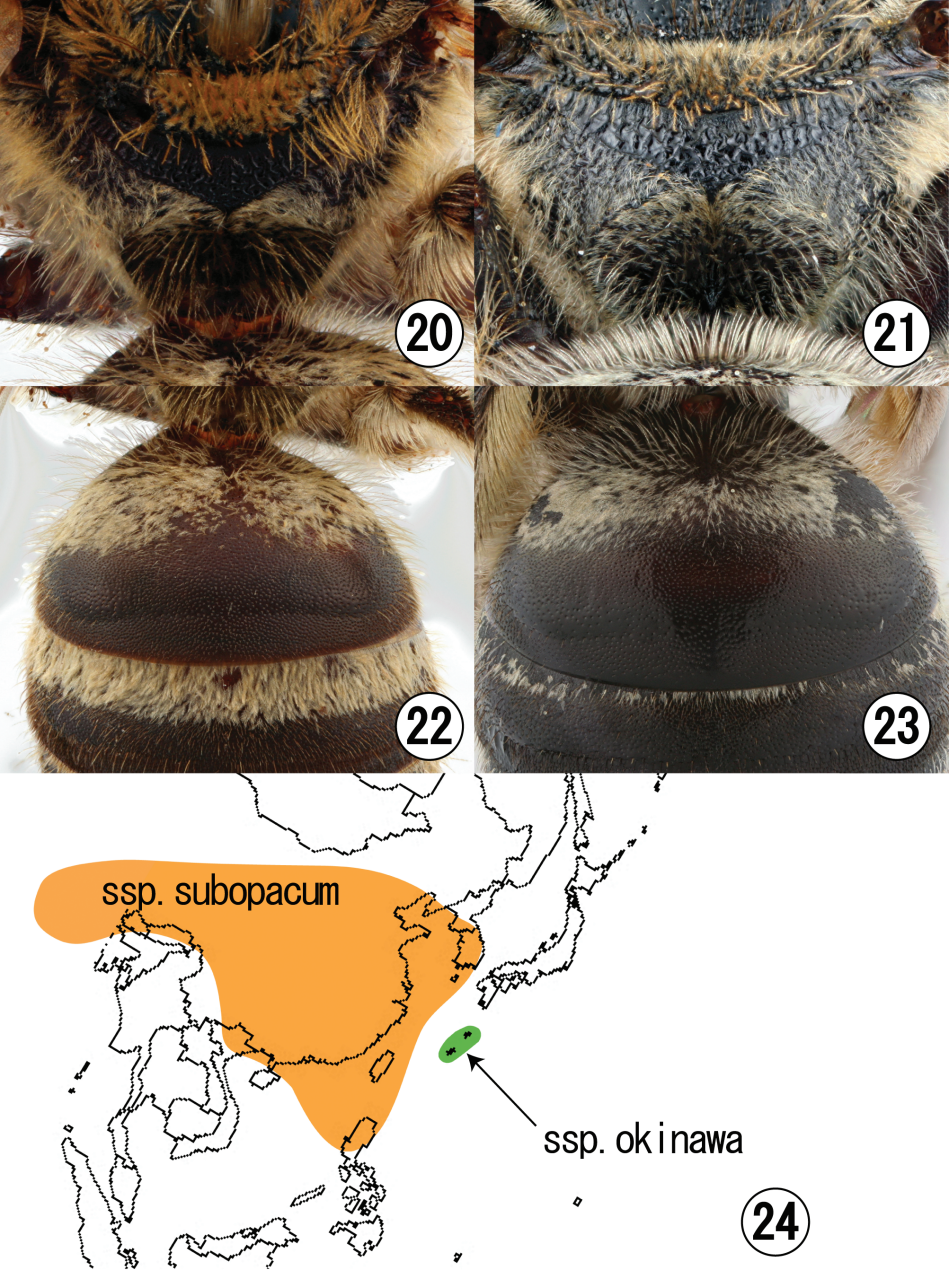
**20, 22** Female of *Lasioglossum (Lasioglossum) subopacum subopacum* (Smith) **21, 23** Female of *Lasioglossum (Lasioglossum) subopacum okinawa* Ebmer et Maeta **20, 21** hairs on propodeum **22, 23** hairs on first metasomal tergum **24** Distribution of both ssp. *subopacum* and ssp. *okinawa*.

## Supplementary Material

XML Treatment for Lasioglossum (Lasioglossum) subopacum subopacum

XML Treatment for Lasioglossum (Lasioglossum) subopacum okinawa
